# 3′-(3-Hy­droxy­phen­yl)-4-methyl­spiro­[benzo[*e*][1,4]diazepine-3,2′-oxirane]-2,5(1*H*,4*H*)-dione

**DOI:** 10.1107/S1600536811022161

**Published:** 2011-06-18

**Authors:** Jun-Liang Liu, Zhi-Yu Hu, Qing-Yan Xu

**Affiliations:** a Laboratory of Microbial Pharmaceutical Engineering, School of Life Sciences, Xiamen University, Xiamen 361005, People’s Republic of China

## Abstract

In the title compound, C_17_H_14_N_2_O_4_, the seven-membered ring adopts a boat conformation, and the two benzene rings make a dihedral angle of 45.22 (5)°. The crystal packing is stabilized by inter­molecular N—H⋯O and O—H⋯O hydrogen bonds.

## Related literature

For the biological activity of the title compound, see: Birkinshaw *et al.* (1963 ▶); Cutler *et al.* (1984 ▶); Heguy, *et al.* (1998 ▶). For the biosynthesis of cyclo­penol, see: Nover & Luckner (1969 ▶).
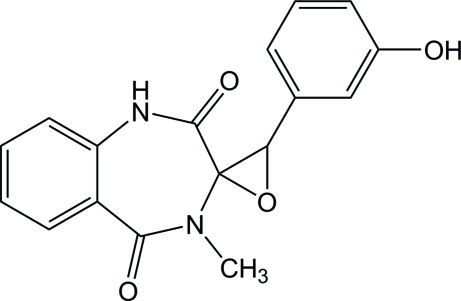


## Experimental

### 

#### Crystal data

C_17_H_14_N_2_O_4_
*M* *_r_* = 310.30
Monoclinic, 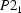

*a* = 7.0066 (2) Å
*b* = 11.6160 (4) Å
*c* = 9.1568 (2) Å
β = 108.157 (1)°
*V* = 708.15 (4) Å^3^
*Z* = 2
Mo *K*α radiation
μ = 0.11 mm^−1^
*T* = 193 K
0.55 × 0.32 × 0.22 mm

#### Data collection

Bruker SMART 1000 CCD area-detector diffractometer
6890 measured reflections
1701 independent reflections
1627 reflections with *I* > 2σ(*I*)
*R*_int_ = 0.021

#### Refinement

*R*[*F* ^2^ > 2σ(*F* ^2^)] = 0.033
*wR*(*F* ^2^) = 0.107
*S* = 0.99
1701 reflections
208 parameters
1 restraint
H-atom parameters constrained
Δρ_max_ = 0.21 e Å^−3^
Δρ_min_ = −0.24 e Å^−3^

### 

Data collection: *SMART* (Bruker, 2007 ▶); cell refinement: *SAINT* (Bruker, 2007 ▶); data reduction: *SAINT*; program(s) used to solve structure: *SHELXS97* (Sheldrick, 2008 ▶); program(s) used to refine structure: *SHELXL97* (Sheldrick, 2008 ▶); molecular graphics: *ORTEP-3* (Farrugia, 1997 ▶); software used to prepare material for publication: *SHELXL97*.

## Supplementary Material

Crystal structure: contains datablock(s) I, global. DOI: 10.1107/S1600536811022161/xu5236sup1.cif

Structure factors: contains datablock(s) I. DOI: 10.1107/S1600536811022161/xu5236Isup2.hkl

Supplementary material file. DOI: 10.1107/S1600536811022161/xu5236Isup3.cml

Additional supplementary materials:  crystallographic information; 3D view; checkCIF report

## Figures and Tables

**Table 1:** Hydrogen-bond geometry (Å, °)

*D*—H⋯*A*	*D*—H	H⋯*A*	*D*⋯*A*	*D*—H⋯*A*
N1—H1⋯O2^i^	0.86	2.13	2.893 (2)	148
O4—H4*A*⋯O1^ii^	0.82	1.95	2.7689 (17)	173

Symmetry codes: (i) 
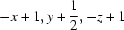
; (ii) 
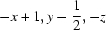
.

**Fig. 1.**
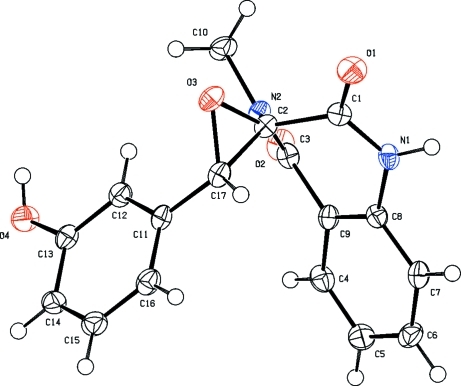
The molecular structure of the title compound with 50% probability ellipsoids.

**Fig. 2.**
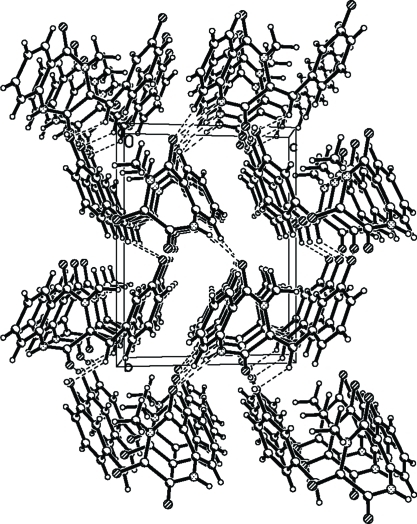
Partial packing diagram of the title compound, viewed along the a axis.

## supplementary crystallographic information

### Comment

The title compund named cyclopenol is an benzodiazepine metabolite produced by a number of *Penicllium* species. Cyclopenol isolated from *Penicillium cyclopium* Westling (Birkinshaw *et al.*, 1963). It display intermediate in the biosynthesis of viridicatins, inhibitors of the TNF-α-induced replication of human HIV (Nover & Luckner, 1969; Heguy, *et al.*, 1998). It is of ecological significance due to its phytotoxic and antimicrobial properties (Cutler, *et al.*, 1984). In our study, we determined the crystal structure of the title compound. The seven membered ring adopts a boat conformation. The crystal packing is stabilized by intermolecular N—H···O and O—H···O hydrogen bondings (Table 1).

### Experimental

The fungal F00734 was cultured using half sea-water Potato Dextrose Agar medium at 28 degrees celsius for 14 days. The fermentation (10 liters) was extracted with ethyl acetate (EtOAc). The EtOAc extract (5.0 g) which was subjected to column chromatography over RP-18, eluted with Methanol-H_2_O (30%, 50%, 70%, 100%; V/V) to yield 10 fractions. Fraction 5, eluted with methanol was further puried by Sephadex LH-20 chromatography. Then merged the components 1–15 tubes eluted with acetone, and further purified by silica-gel column chromatography to afford the title compound 1 (140.0 mg). F00734 is an high-yield strain of cyclopenol (2.8%).

### Refinement

H atoms were positioned geometrically and were treated as riding on their parent atoms, with C—H distances of 0.93–0.98 Å, an N—H distance of 0.86 Å, and O—H distance of 0.82Å and *U*_iso_(H)=1.2U_eq_(N,C).

### Crystal data

**Table:**

C_17_H_14_N_2_O_4_	*F*(000) = 324
*M**_r_* = 310.30	*D*_x_ = 1.455 Mg m^−^^3^
Monoclinic, *P*2_1_	Mo *K*α radiation, λ = 0.71073 Å
Hall symbol: P 2yb	Cell parameters from 1484 reflections
*a* = 7.0066 (2) Å	θ = 1.9–27.5°
*b* = 11.6160 (4) Å	µ = 0.11 mm^−^^1^
*c* = 9.1568 (2) Å	*T* = 193 K
β = 108.157 (1)°	Block, colourless
*V* = 708.15 (4) Å^3^	0.55 × 0.32 × 0.22 mm
*Z* = 2	

### Data collection

**Table:**

Bruker SMART 1000 CCD area-detector diffractometer	1627 reflections with *I* > 2σ(*I*)
Radiation source: fine-focus sealed tube	*R*_int_ = 0.021
graphite	θ_max_ = 27.5°, θ_min_ = 3.1°
φ and ω scans	*h* = −8→9
6890 measured reflections	*k* = −15→15
1701 independent reflections	*l* = −11→11

### Refinement

**Table:**

Refinement on *F*^2^	Primary atom site location: structure-invariant direct methods
Least-squares matrix: full	Secondary atom site location: difference Fourier map
*R*[*F*^2^ > 2σ(*F*^2^)] = 0.033	Hydrogen site location: inferred from neighbouring sites
*wR*(*F*^2^) = 0.107	H-atom parameters constrained
*S* = 0.99	*w* = 1/[σ^2^(*F*_o_^2^) + (0.1*P*)^2^] where *P* = (*F*_o_^2^ + 2*F*_c_^2^)/3
1701 reflections	(Δ/σ)_max_ = 0.009
208 parameters	Δρ_max_ = 0.21 e Å^−^^3^
1 restraint	Δρ_min_ = −0.24 e Å^−^^3^

### Special details

**Table:**

Geometry. All e.s.d.'s (except the e.s.d. in the dihedral angle between two l.s. planes) are estimated using the full covariance matrix. The cell e.s.d.'s are taken into account individually in the estimation of e.s.d.'s in distances, angles and torsion angles; correlations between e.s.d.'s in cell parameters are only used when they are defined by crystal symmetry. An approximate (isotropic) treatment of cell e.s.d.'s is used for estimating e.s.d.'s involving l.s. planes.
Refinement. Refinement of *F*^2^ against ALL reflections. The weighted *R*-factor *wR* and goodness of fit *S* are based on *F*^2^, conventional *R*-factors *R* are based on *F*, with *F* set to zero for negative *F*^2^. The threshold expression of *F*^2^ > σ(*F*^2^) is used only for calculating *R*-factors(gt) *etc*. and is not relevant to the choice of reflections for refinement. *R*-factors based on *F*^2^ are statistically about twice as large as those based on *F*, and *R*- factors based on ALL data will be even larger.

### Fractional atomic coordinates and isotropic or equivalent isotropic displacement parameters (Å^2^)

**Table:**

	*x*	*y*	*z*	*U*_iso_*/*U*_eq_	
O2	0.4377 (2)	0.08072 (14)	0.31423 (17)	0.0269 (3)	
O3	0.4521 (2)	0.40192 (14)	0.02123 (17)	0.0227 (3)	
O1	0.6694 (2)	0.51497 (15)	0.29249 (19)	0.0291 (4)	
O4	−0.0361 (2)	0.06229 (14)	−0.25108 (18)	0.0271 (4)	
H4A	0.0758	0.0542	−0.2604	0.041*	
N2	0.4837 (2)	0.24388 (15)	0.19567 (18)	0.0184 (3)	
N1	0.4887 (2)	0.42631 (15)	0.42617 (18)	0.0205 (4)	
H1	0.5578	0.4610	0.5087	0.025*	
C8	0.3244 (3)	0.35791 (17)	0.4353 (2)	0.0186 (4)	
C9	0.2804 (3)	0.24831 (18)	0.3691 (2)	0.0186 (4)	
C7	0.2100 (3)	0.40240 (19)	0.5227 (2)	0.0216 (4)	
H7	0.2433	0.4733	0.5712	0.026*	
C4	0.1162 (3)	0.18798 (19)	0.3878 (2)	0.0236 (4)	
H4	0.0858	0.1152	0.3445	0.028*	
C3	0.4063 (3)	0.18510 (18)	0.2913 (2)	0.0192 (4)	
C12	0.1144 (3)	0.22949 (19)	−0.1034 (2)	0.0212 (4)	
H12	0.2423	0.1976	−0.0787	0.025*	
C10	0.6209 (3)	0.1863 (2)	0.1253 (2)	0.0257 (4)	
H10A	0.6350	0.1068	0.1554	0.038*	
H10B	0.7499	0.2231	0.1592	0.038*	
H10C	0.5671	0.1915	0.0154	0.038*	
C16	−0.1031 (3)	0.3876 (2)	−0.0884 (2)	0.0241 (4)	
H16	−0.1207	0.4607	−0.0531	0.029*	
C11	0.0875 (3)	0.33865 (18)	−0.0492 (2)	0.0201 (4)	
C14	−0.2433 (3)	0.21769 (19)	−0.2334 (2)	0.0234 (4)	
H14	−0.3541	0.1771	−0.2941	0.028*	
C6	0.0469 (3)	0.3415 (2)	0.5378 (2)	0.0247 (4)	
H6	−0.0307	0.3725	0.5941	0.030*	
C13	−0.0508 (3)	0.16860 (19)	−0.1948 (2)	0.0208 (4)	
C17	0.2579 (3)	0.40891 (18)	0.0485 (2)	0.0198 (4)	
H17	0.2200	0.4869	0.0695	0.024*	
C15	−0.2672 (3)	0.3264 (2)	−0.1808 (2)	0.0252 (4)	
H15	−0.3946	0.3592	−0.2075	0.030*	
C2	0.4422 (3)	0.36451 (16)	0.1645 (2)	0.0180 (4)	
C5	−0.0007 (3)	0.2341 (2)	0.4688 (2)	0.0267 (5)	
H5	−0.1113	0.1937	0.4774	0.032*	
C1	0.5463 (3)	0.44186 (18)	0.2995 (2)	0.0195 (4)	

### Atomic displacement parameters (Å^2^)

**Table:**

	*U*^11^	*U*^22^	*U*^33^	*U*^12^	*U*^13^	*U*^23^
O2	0.0382 (8)	0.0157 (7)	0.0268 (7)	0.0038 (6)	0.0100 (6)	0.0041 (6)
O3	0.0288 (7)	0.0214 (7)	0.0222 (7)	0.0002 (5)	0.0139 (5)	0.0024 (6)
O1	0.0309 (8)	0.0236 (8)	0.0359 (8)	−0.0097 (6)	0.0149 (6)	−0.0027 (7)
O4	0.0268 (7)	0.0222 (8)	0.0341 (8)	−0.0028 (6)	0.0121 (6)	−0.0060 (6)
N2	0.0227 (7)	0.0155 (8)	0.0180 (7)	0.0029 (6)	0.0077 (6)	0.0006 (7)
N1	0.0236 (8)	0.0174 (8)	0.0189 (8)	−0.0052 (6)	0.0044 (6)	−0.0034 (6)
C8	0.0194 (9)	0.0184 (9)	0.0169 (8)	−0.0022 (7)	0.0042 (7)	0.0015 (7)
C9	0.0225 (8)	0.0181 (9)	0.0144 (8)	0.0006 (7)	0.0046 (7)	0.0022 (7)
C7	0.0273 (9)	0.0183 (9)	0.0180 (8)	0.0001 (7)	0.0052 (7)	−0.0012 (7)
C4	0.0251 (9)	0.0204 (10)	0.0234 (9)	−0.0063 (8)	0.0050 (7)	0.0004 (8)
C3	0.0220 (9)	0.0140 (9)	0.0188 (8)	0.0025 (7)	0.0021 (6)	0.0016 (7)
C12	0.0234 (9)	0.0216 (10)	0.0186 (8)	0.0041 (7)	0.0065 (7)	0.0022 (7)
C10	0.0248 (10)	0.0251 (11)	0.0290 (9)	0.0094 (8)	0.0113 (7)	−0.0007 (8)
C16	0.0289 (10)	0.0229 (10)	0.0216 (9)	0.0058 (8)	0.0096 (8)	0.0013 (8)
C11	0.0256 (9)	0.0208 (10)	0.0149 (8)	0.0012 (7)	0.0076 (7)	0.0007 (7)
C14	0.0204 (9)	0.0291 (12)	0.0213 (9)	−0.0014 (7)	0.0071 (7)	0.0018 (8)
C6	0.0277 (10)	0.0291 (11)	0.0202 (9)	0.0014 (8)	0.0117 (8)	0.0027 (8)
C13	0.0265 (9)	0.0187 (9)	0.0195 (8)	−0.0010 (8)	0.0105 (7)	0.0000 (7)
C17	0.0263 (10)	0.0163 (9)	0.0178 (8)	0.0017 (7)	0.0085 (7)	0.0028 (7)
C15	0.0234 (10)	0.0300 (12)	0.0230 (10)	0.0054 (8)	0.0082 (8)	0.0030 (8)
C2	0.0220 (9)	0.0143 (9)	0.0193 (8)	0.0005 (7)	0.0087 (7)	0.0016 (7)
C5	0.0263 (10)	0.0300 (12)	0.0252 (10)	−0.0084 (9)	0.0101 (8)	0.0025 (9)
C1	0.0200 (8)	0.0149 (9)	0.0238 (9)	0.0001 (6)	0.0069 (7)	−0.0002 (7)

### Geometric parameters (Å, °)

**Table:**

O2—C3	1.238 (3)	C12—C13	1.392 (3)
O3—C2	1.405 (2)	C12—C11	1.396 (3)
O3—C17	1.461 (2)	C12—H12	0.9300
O1—C1	1.226 (3)	C10—H10A	0.9600
O4—C13	1.354 (3)	C10—H10B	0.9600
O4—H4A	0.8200	C10—H10C	0.9600
N2—C3	1.350 (3)	C16—C15	1.392 (3)
N2—C2	1.441 (2)	C16—C11	1.392 (3)
N2—C10	1.474 (2)	C16—H16	0.9300
N1—C1	1.354 (3)	C11—C17	1.492 (3)
N1—C8	1.422 (2)	C14—C15	1.380 (3)
N1—H1	0.8600	C14—C13	1.404 (3)
C8—C7	1.397 (3)	C14—H14	0.9300
C8—C9	1.402 (3)	C6—C5	1.391 (3)
C9—C4	1.403 (3)	C6—H6	0.9300
C9—C3	1.489 (3)	C17—C2	1.486 (3)
C7—C6	1.387 (3)	C17—H17	0.9800
C7—H7	0.9300	C15—H15	0.9300
C4—C5	1.373 (3)	C2—C1	1.518 (3)
C4—H4	0.9300	C5—H5	0.9300
			
C2—O3—C17	62.43 (12)	C11—C16—H16	120.2
C13—O4—H4A	109.5	C16—C11—C12	120.29 (19)
C3—N2—C2	121.51 (17)	C16—C11—C17	117.10 (18)
C3—N2—C10	120.16 (17)	C12—C11—C17	122.59 (17)
C2—N2—C10	118.28 (17)	C15—C14—C13	119.7 (2)
C1—N1—C8	125.99 (16)	C15—C14—H14	120.1
C1—N1—H1	117.0	C13—C14—H14	120.1
C8—N1—H1	117.0	C7—C6—C5	119.99 (19)
C7—C8—C9	119.68 (18)	C7—C6—H6	120.0
C7—C8—N1	116.53 (18)	C5—C6—H6	120.0
C9—C8—N1	123.70 (17)	O4—C13—C12	122.98 (18)
C4—C9—C8	118.62 (18)	O4—C13—C14	117.11 (18)
C4—C9—C3	116.31 (19)	C12—C13—C14	119.9 (2)
C8—C9—C3	124.83 (17)	O3—C17—C2	56.95 (11)
C6—C7—C8	120.4 (2)	O3—C17—C11	118.78 (16)
C6—C7—H7	119.8	C2—C17—C11	126.41 (18)
C8—C7—H7	119.8	O3—C17—H17	114.1
C5—C4—C9	121.4 (2)	C2—C17—H17	114.1
C5—C4—H4	119.3	C11—C17—H17	114.1
C9—C4—H4	119.3	C14—C15—C16	120.80 (18)
O2—C3—N2	121.27 (19)	C14—C15—H15	119.6
O2—C3—C9	120.14 (19)	C16—C15—H15	119.6
N2—C3—C9	118.58 (18)	O3—C2—N2	114.81 (16)
C13—C12—C11	119.76 (18)	O3—C2—C17	60.63 (12)
C13—C12—H12	120.1	N2—C2—C17	123.77 (17)
C11—C12—H12	120.1	O3—C2—C1	115.22 (16)
N2—C10—H10A	109.5	N2—C2—C1	113.46 (16)
N2—C10—H10B	109.5	C17—C2—C1	117.85 (17)
H10A—C10—H10B	109.5	C4—C5—C6	119.79 (19)
N2—C10—H10C	109.5	C4—C5—H5	120.1
H10A—C10—H10C	109.5	C6—C5—H5	120.1
H10B—C10—H10C	109.5	O1—C1—N1	122.65 (19)
C15—C16—C11	119.5 (2)	O1—C1—C2	122.34 (19)
C15—C16—H16	120.2	N1—C1—C2	114.97 (17)

### Hydrogen-bond geometry (Å, °)

**Table:**

*D*—H···*A*	*D*—H	H···*A*	*D*···*A*	*D*—H···*A*
N1—H1···O2^i^	0.86	2.13	2.893 (2)	148
O4—H4A···O1^ii^	0.82	1.95	2.7689 (17)	173

Symmetry codes: (i) −*x*+1, *y*+1/2, −*z*+1; (ii) −*x*+1, *y*−1/2, −*z*.
